# Microstructure and Mechanical Properties of Friction Stir Welded the Novel Al-Mg-Zn-Si Alloy

**DOI:** 10.3390/ma18235269

**Published:** 2025-11-21

**Authors:** Junzhe Huang, Ying Li, Xiwu Li, Hongwei Yan, Lizhen Yan, Kai Wen, Yanan Li, Guanjun Gao, Kai Zhu, Mingyang Yu, Yongan Zhang, Baiqing Xiong

**Affiliations:** 1 State Key Laboratory of Nonferrous Structural Materials, China GRINM Group Co., Ltd., Beijing 100088, China; 2 GRIMAT Engineering Institute Co., Ltd., Beijing 101407, China; 3 General Research Institute for Nonferrous Metals, Beijing 100088, China

**Keywords:** Al-Mg-Zn-Si cross alloy, high Mg-containing aluminum alloy, friction stir welding, microstructure, mechanical properties

## Abstract

The high-Mg-content Al-Mg-Zn-Si alloy, as a novel aluminum alloy, exhibits excellent strength, toughness, and corrosion resistance, demonstrating significant application potential in lightweight structural components for aerospace, weapon systems, rail transportation, and other fields. In this study, friction stir welding was employed to weld the high-Mg-content Al-Mg-Zn-Si alloy. Subsequent aging treatment was applied to establish the relationship between the mechanical properties and microstructural characteristics of the welded joint, aiming to elucidate the strengthening mechanisms of the new alloy and provide insights for achieving high-quality welds. The results indicate that the microhardness profile of the as-welded joint exhibited a “W” shape, with overall low hardness values and minor differences between zones. After the aging treatment, the microhardness increased significantly in the base material (BM), the thermo-mechanically affected zone (TMAZ), and the stir zone (SZ), whereas the heat-affected zone (HAZ) adjacent to the SZ exhibited only a marginal increase, making it the softest region in the aged joint. The yield strength and ultimate tensile strength of the aged joint increased to 327 MPa and 471 MPa, respectively. The enhancement in microhardness and strength after aging treatment was attributed to the precipitation of numerous nano-sized T-phase particles within grains. Interestingly, the tensile samples of the aged joint fractured in the high-hardness SZ instead of the low-hardness HAZ. This fracture behavior was primarily attributed to continuous grain boundary precipitates, which reduced intergranular cohesion. In contrast, the elongated grain structure in the HAZ more effectively resisted intergranular crack propagation compared to the equiaxed grains in the SZ.

## 1. Introduction

Structural lightweighting is a perpetual theme in the development of fields such as aviation, aerospace, weaponry, and rail transportation [1,2]. Aluminum alloys are widely used in the manufacturing of various lightweight structural components due to their low density (~2.7 g/cm^3^), ease of processing, recyclability, and abundant raw material resources [3]. For example, the 7xxx series (Al-Zn-Mg-Cu) high-strength aluminum alloys [4,5], 2xxx series (Al-Cu-Mg) medium–high-strength damage-tolerant aluminum alloys [6], and 6xxx series (Al-Mg-Si) medium–high-strength aluminum alloys serve as the backbone lightweight structural materials for aerospace vehicles, weaponry, and high-speed trains [7,8]. However, with the increasing demand for enhanced performance in aerospace vehicles, weaponry, and high-speed trains, further weight reduction in structural components is required [9]. The widespread industrial application of third-generation Al-Li alloys, coupled with intensive ongoing research into fourth-generation variants, demonstrates how materials with high specific strength and reduced density effectively address lightweighting requirements in structural applications. Consequently, developing new aluminum alloys with excellent comprehensive properties and lower density is an effective approach to achieving weight reduction in structural components [10].

Magnesium (Mg) is one of the primary alloying elements in aluminum alloys, with a density of only 1.74 g/cm^3^ [11]. Each 1 wt% increase in Mg content reduces the density of aluminum alloys by 0.38%, making it highly beneficial for lowering the density of aluminum alloys. However, excessive addition of Mg beyond the alloy’s maximum solid solubility can lead to the precipitation of a network-like second phase at grain boundaries, severely degrading the comprehensive properties of the alloy [12]. Xiong et al. [13] innovatively developed a new high-Mg-content Al-Mg-Zn-Si alloy by adding Zn and Si elements to neutralize the excess Mg. With a lower density of 2.62 g/cm^3^ and higher ultimate tensile strength (625 MPa, peak-aged), this alloy demonstrates superior specific properties compared to 7055-T7751 (614 MPa, 2.86 g/cm^3^). This approach reduces the segregation of Mg-containing phases at grain boundaries while transforming the surplus Mg into precipitation-strengthening phases (T-Mg_32_(AlZn)_49_), thereby enhancing the alloy’s strength [14]. This new alloy exhibits excellent strength, toughness, and corrosion resistance, showing great application potential in lightweight structural components used in aerospace, weaponry, rail transportation, and other fields [15]. However, systematic research on the comprehensive properties of this new alloy, such as its welding performance, remains to be conducted.

In the novel aluminum alloy, the incorporation of Mg and Zn elements renders it susceptible to the formation of low-melting-point eutectic structures along grain boundaries during conventional fusion welding, thereby promoting liquation cracking. Furthermore, the burning loss of elements, such as Mg and Zn, during the welding process often facilitates the formation of metallurgical porosity, which significantly compromises the service stability of the welded joint. Friction stir welding (FSW) effectively circumvents the typical defects inherent in fusion welding methods, as the material within the weld zone remains in the solid state throughout the process, thereby avoiding melting and subsequent solidification [16,17]. FSW technology has been successfully applied in the field of aluminum alloy welding, and the performance of the welded joints is significantly superior to that of traditional fusion-welded joints [18]. Numerous studies have been reported on the FSW technology for medium- and low-Mg-content 5xxx aluminum alloys [19,20], but research on the FSW of high-Mg-content 5xxx aluminum alloys is rarely reported. Considering that the Mg content of the new Al-Mg-Zn-Si alloy can be as high as 6–8 wt.%, and Mg can significantly reduce the stacking fault energy of the aluminum matrix [21], if the new alloy is subjected to friction stir welding, it is likely to induce intense plastic deformation and dynamic recrystallization in the welded joint, thereby refining the grain structure of the joint and improving its strength and plasticity [22]. Furthermore, due to the addition of Zn and Si elements in the new alloy, precipitation-strengthening phases are formed [23,24,25]. During the FSW process, the strengthening phases in different regions of the joint will undergo varying degrees of dissolution, reprecipitation, and coarsening, affecting the mechanical properties of the welded joint [26,27].

Therefore, this study focused on friction stir welding of a novel Al-Mg-Zn-Si alloy, systematically investigating the microstructural characteristics of different regions of the welded joint and their correlation with the mechanical properties. This is of great significance for promoting the development and application of the new alloy.

## 2. Materials and Experiments

### 2.1. Materials and FSW Process

**Figure 1:**
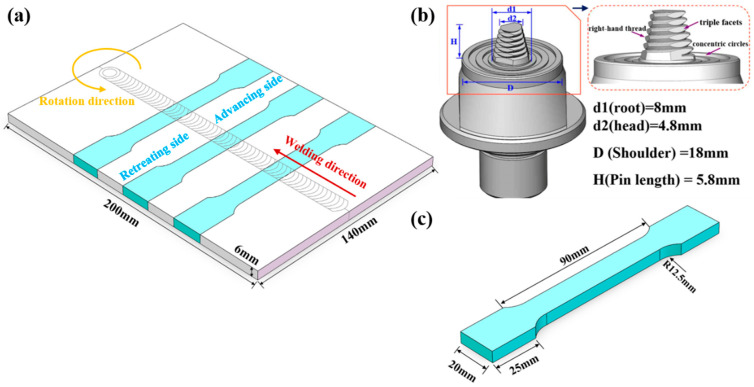
Schematic of the welding process and diagram of tensile specimen: (**a**) schematic diagram of welding, (**b**) the structure of the welding tool, and (**c**) schematic diagram of tensile specimen (unit: mm).

This study used 6 mm thick Al-Mg-Zn-Si aluminum alloy plate in a natural aging state as the base material (BM). The chemical composition of the BM is 6.0–8.0 wt.% Mg, 2.0–4.0 wt.% Zn, and 0–1.0 wt.% Si, and the balance is Al. Before the FSW process, all the plates were cleaned with ethanol. During the FSW process, the optimal FSW parameters, with 500 r/min rotation speed and 100 mm/min welding speed, were used for welding. The schematic diagrams of the FSW process and the welding tool used in this study were shown in Figure 1a,b. The novel alloy was friction stir welded using a model HT-JM16x15/2 gantry-type 2-axis FSW system (AEE, Suzhou, China). The detailed tool parameters are provided in Figure 1b. The welding was performed with a plunge depth of 0.2 mm, a tool tilt angle of 2.5°, and a dwell time of 10 s.

After FSW process, the microhardness, tensile properties, and microstructures of the joints were tested in the as-welded state and artificial aging state. The aged samples were subjected to aging in a forced-air drying oven at 90 °C for 24 h, followed by further aging at 115 °C for 15 h.

### 2.2. Microhardness and Tensile Test

The microhardness distribution on the cross-section of the joints in the as-welded and artificial aged states were tested using Vickers microhardness tester (Buehler Wilson VH1150, Lake Bluff, IL, USA) with a 5 kg load and 1 mm spacing between indentations (total of 71 measurements, distributed as follows: 5 in the SZ, 6 across both sides of the TMAZ, 36 across both sides of the HAZ, and 24 in the BM on both sides), to characterize the hardness profile.

The tensile samples were taken from the joint perpendicular to the welding direction. The sampling diagram and dimensions of the tensile samples are shown in Figure 1a and Figure 1c, respectively. Tensile specimens with a gauge length of 50 mm were cut to the welding direction, in accordance with the ASTM E8-04 [28]. The tensile properties of the joints in the as-welded and artificial aged states were tested at room temperature using a CMT5105 universal testing machine (SUNS, Shenzhen, China) with a constant crosshead speed of 2 mm/min and tests were repeated three times to minimize experimental variability.

### 2.3. Microstructural Observations

Microstructures including fractography, grain structures, and precipitate features in different regions of the joints were observed using an optical microscope (OM, Leica-DMi8C, Wetzlar, Germany), a scanning electron microscope (SEM, JSM-7900F, JEOL Ltd., Tokyo, Japan), an electron backscatter diffraction (EBSD, JSM-7900F) and a transmission electron microscopy (TEM, Talos F200X, Thermo Fisher Scientific, Waltham, MA, USA). The fractograph of the fracture surface was obtained using SEM. The grain structures were characterized by EBSD using a step size of 0.1 μm and a confidence index (CI) threshold of 0.90, and the EBSD samples were electropolished in a solution comprising 10 vol.% perchloric acid and 90 vol.% methanol for 5 s at −10 °C and 20 V. The characteristics of the particles were explored using scanning TEM (STEM) combined with energy-dispersive X-ray spectroscopy (EDS). High-angle annular dark-field scanning transmission electron microscopy (HAADF-STEM, Thermo Fisher Scientific, Waltham, MA, USA) was additionally employed for microchemical composition analysis through both point and elemental mapping. The TEM samples were electropolished using a twin-jet system with a solution of 25% nitric acid in methanol at −30 to −20 °C and an applied voltage of 15–20 V.

## 3. Results

### 3.1. Microhardness

**Figure 2:**
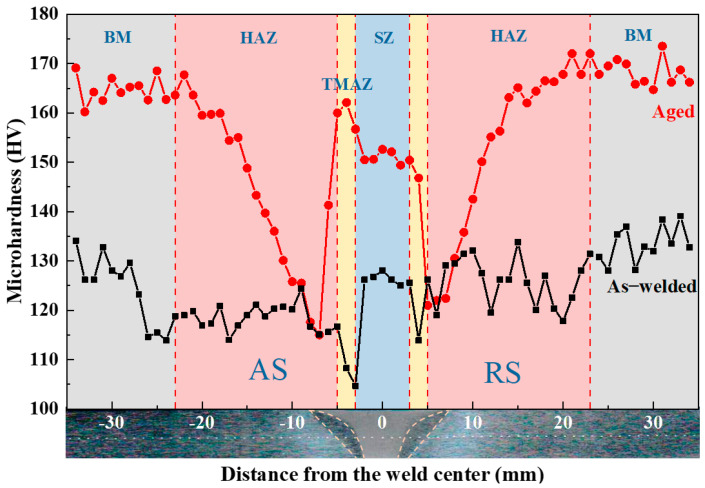
Microhardness profiles of the as-welded and aged (90 °C/24 h + 115 °C/15 h) test joints.

Figure 2 presents the microhardness distribution of the cross-section of the as-welded and aged joints. In the as-welded state, the microhardness values in the stir zone (SZ), thermo-mechanically affected zone (TMAZ), and heat-affected zone (HAZ) were slightly lower than that of BM. The lowest microhardness occurred in the SZ near the TMAZ. The microhardness distribution following the artificial aging treatment at 90 °C/24 h + 115 °C/15 h was characterized by a distinct W-shaped profile. The microhardness in the SZ increased from 125 HV to 150 HV, and that in the TMAZ rose from 110 HV to 155 HV. The microhardness showed little increase in the HAZ adjacent to the SZ, whereas the BM demonstrated an increase from 130 HV to 165 HV. The average microhardness value in SZ and TMAZ of aged joint reached approximately 90% of that of the BM.

### 3.2. Tensile Properties

**Figure 3:**
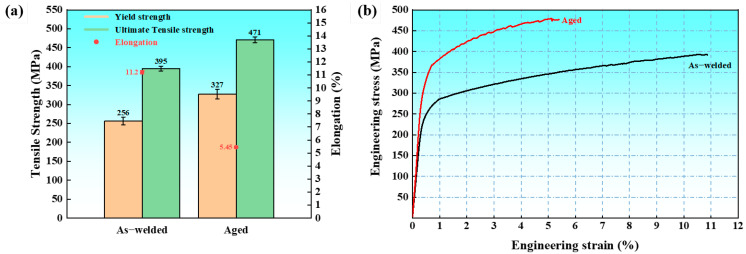
Tensile properties of the as-welded and aged joints: (**a**) the yield strength, ultimate tensile strength, and elongation of the joints, and (**b**) the stress–strain curves of the joints.

Figure 3a compared the tensile properties of the as-welded and aged joints. The corresponding engineering stress–strain curves of the joints are shown in Figure 3b. The average yield strength, ultimate tensile strength, and elongation of the as-welded joint were 256 MPa, 395 MPa, and 11.20%, respectively. After the aging treatment in conditions of 90 °C/24 h + 115 °C/15 h, the average yield strength and ultimate tensile strength increased to 327 MPa and 471 MPa, respectively; however, the elongation of the aged joint decreased to 5.45%. The tensile property results revealed that the joint strength increased by over 70 MPa after the aging treatment; however, the aging treatment resulted in a 50% reduction in elongation compared to the as-welded joint. Moreover, the aged joint achieved 75% of the base material’s ultimate tensile strength (625 MPa).

**Figure 4:**
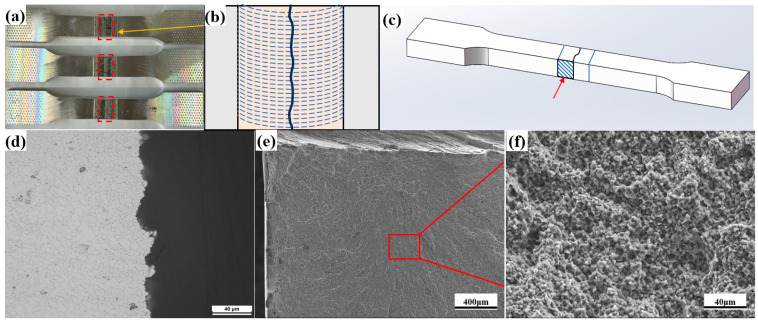
Fracture characteristics of the tensile samples of the aged joint: (**a**) macroscopic diagram of tensile samples, (**b**) schematic diagram of the fracture location, (**c**) schematic diagram of metallographic sample extraction, (**d**) optical microscopy on the cross-section of the tensile sample, and (**e**,**f**) SEM images of the fracture surface from a tensile sample.

The FSW joints both in the as-welded and aged state fractured in the SZ during tensile testing. Figure 4a,b present the macrograph of the fractured tensile samples and schematic diagram of the fracture location, respectively. This finding contrasted with the microhardness profile, where significantly elevated hardness values were recorded in the SZ, particularly after aging treatment. Microstructural analyses of tensile specimen cross-sections (Figure 4c,d) and fracture morphologies (Figure 4e,f) were performed to detect the reason of apparent contradiction between the fracture localization and microhardness distribution profiles in the welded joints. Both OM and SEM analyses confirmed that the tensile samples failed via an intergranular fracture mode. The fact that fracture occurred in the high-hardness SZ of defect-free welds suggests a weakening of the grain boundaries, which facilitated preferential crack propagation during plastic deformation.

### 3.3. Microstructure

**Figure 5:**
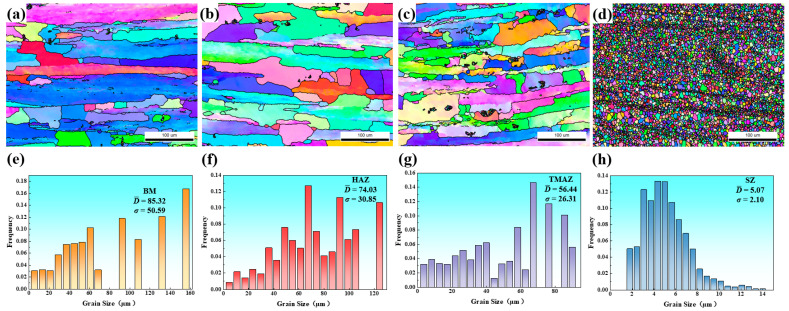
Inverse pole figure (IPF) maps and the corresponding grain size distributions of different regions in the as-welded joint: (**a**,**e**) BM, (**b**,**f**) HAZ, (**c**,**g**) TMAZ, and (**d**,**h**) SZ.

Figure 5 presents the inverse pole figure (IPF) maps and the corresponding grain size distributions of different regions in the as-welded joint. Figure 5a shows elongated grain morphology in the BM region, with an average grain size of 85.22 μm (Figure 5e). During the FSW process, the SZ underwent dynamic recrystallization due to the intense thermo-mechanical effect, resulting in fine equiaxed grains forming in the SZ (Figure 5d). The average grain size in the SZ was reduced to 5.07 μm (Figure 5h). In comparison to the SZ, the TMAZ was subjected to insufficient plastic deformation during the FSW process, thereby restricting the extent of grain deformation and dynamic recrystallization (Figure 5c). The average grain size in the TMAZ was measured at 56.44 μm (Figure 5g). The HAZ, only subjected to thermal cycling during welding process, exhibiting static recovery and static recrystallization. While the grain morphology remained elongated (Figure 5b), the grain size reduced to 74.03 μm (Figure 5f).

**Figure 6:**
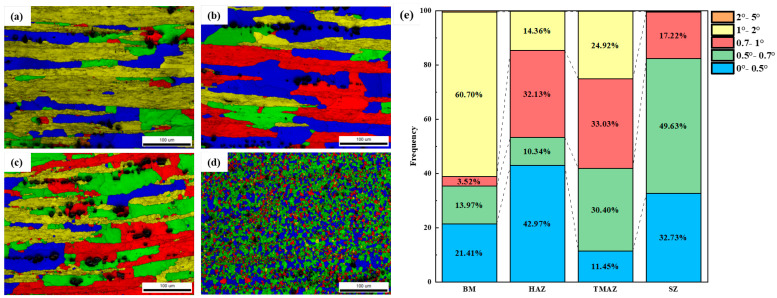
Image quality–grain average misorientation (IQ-GAM) maps and analyze of each zone: (**a**) BM, (**b**) HAZ, (**c**) TMAZ, (**d**) SZ, (**e**) GAM statistical results.

Figure 6 presents the image quality–grain average misorientation (IQ-GAM) maps of each region of the as-welded joint, which reveal the percent of recrystallized grains, sub-grains, and deformed grains. Recrystallized grains exhibit no substructure; therefore, those with misorientation angles below 0.7°, indicated in blue and green, were classified as recrystallized grains [29,30]. Grains with misorientation angles between 0.7° and 2°, marked in red and yellow, were identified as sub-grains, while those exceeding 2° were categorized as deformed grains. The statistical results of the GAM indicated significant differences in the proportion of recrystallized grains across various regions of the as-welded joint, with the following descending order: SZ (82%) > HAZ (53%) > TMAZ (42%) > BM (35%).

**Figure 7:**
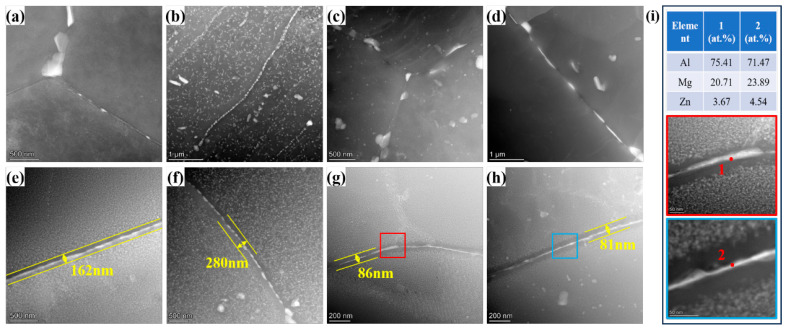
The HAADF images in various regions of the as-welded and aged joints: (**a**) BM of the as-welded joint, (**b**) HAZ of the as-welded joint, (**c**) TMAZ of the as-welded joint, (**d**) SZ of the as-welded joint, (**e**) BM of the aged joint, (**f**) HAZ of the aged joint, and (**g**) TMAZ of the aged joint, (**h**) SZ of the aged joint; (**i**) STEM-EDS results of GPBs in TMAZ and SZ.

Figure 7a–d presents the HAADF images of different regions in the as-welded joint. Microstructural analysis revealed that in the as-welded condition, grain boundary precipitates (GBPs) displayed a discontinuous distribution across the BM, TMAZ, and SZ. Additionally, minimal intragranular precipitates were observed in the BM, TMAZ, and SZ. Due to the thermal exposure in the HAZ during the FSW process, a large number of precipitates formed within the grains and at the grain boundaries. Figure 7e–h shows the HAADF images of different regions in the aged joint. After artificial aging at 90 °C/24 h and 115 °C/15 h, a large number of precipitates were formed both at the grain boundaries and within the grains in the BM, HAZ, TMAZ, and SZ. Moreover, the GPBs exhibited continuous distribution. Precipitation free zones (PFZs) were observed along grain boundaries. In contrast to the BM and HAZ, which developed wider PFZs and larger GBPs, the TMAZ and SZ were characterized by narrower PFZs and finer GBPs. The chemical composition of the GPBs, shown in Figure 7i, revealed that the GPBs contained Al, Zn, and Mg elements. The chemical test results and previous studies based on the Al–Mg–Zn cross alloy [31,32,33] indicated that the GPBs were primarily verified as T-phase.

**Figure 8:**
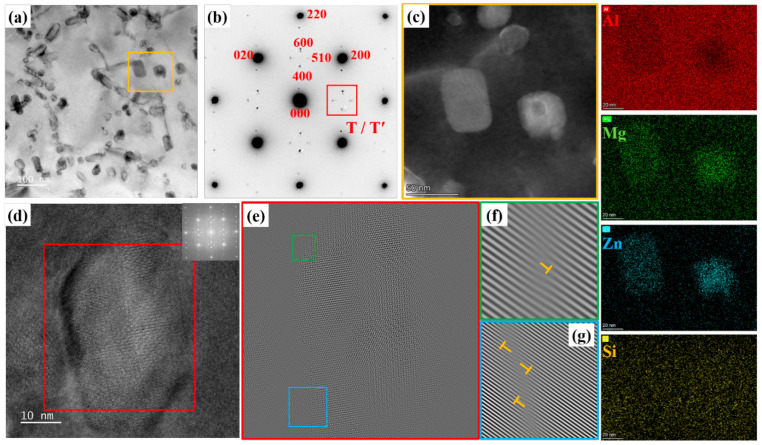
Precipitate characteristics of aged joint: (**a**) bright-field TEM image of HAZ, (**b**) SAED image along <100>_Al_ zone axis, (**c**) HAADF image of the region marked with yellow line in (**a**) an image and the corresponding STEM-EDS mapping images of the Al, Mg, Zn, and Si elements, (**d**) the HRTEM image of the precipitates, and (**e**) the inverse FFT image after mask of the selected precipitate in (**d**) image; (**f**,**g**) diffraction bands with (200)_Al_ planes by inverse FFT extracted from (**d**) image.

To further identify the intragranular precipitates in the aged joint, the HAZ was selected as a representative region for TEM sampling. For this purpose, a comprehensive characterization utilizing TEM, SAED, HRTEM, and FFT was performed. Figure 8a shows the bright-field TEM image of HAZ in the aged joint. The corresponding selected area electron diffraction (SAED) pattern along the <100>_Al_ zone axis shown in Figure 8b reveals the diffraction spots from the T and T′ phases observed at positions 1/3 and 2/3 {022}_Al_. According to the references [34,35,36], the mean precipitates are T-phase. Figure 8c shows the STEM-EDS mappings of the precipitates. It can be clearly seen that the precipitate contains segregation of the Mg and Zn elements without clear segregation of the Si element. It further declares that the precipitates in grains were T-phase. Figure 8d displays the high-resolution transmission electron microscopy (HRTEM) image of the precipitate. Figure 8e is the inverse FFT image after the masking of the selected precipitate in Figure 8d. As demonstrated by the selected area diffraction patterns corresponding to the (200)_Al_ plane in Figure 8f,g, significant lattice distortion is present at the phase–matrix interface. The strain field in this region, denoted by orange symbols, confirms a semi-coherent relationship between the T-phase and the aluminum matrix.

## 4. Discussion

### 4.1. Correlation Between Mechanical Property Evolution and Precipitate Transformation in FSW Joints

Due to the distinct thermo-mechanical effects experienced during the FSW process, the welded joint can be divided into several regions: the SZ, TMAZ, HAZ, and BM. The BM used in this study was a rolled sheet in the T4 condition, exhibiting elongated grains primarily composed of deformed structures (Figure 5a). The precipitation characteristics included the absence of significant intragranular strengthening precipitates, with only a small amount of T-phase distributed along grain boundaries (Figure 7a).

The predominant strengthening mechanisms in this novel alloy comprise precipitation hardening, solid solution strengthening, dislocation strengthening, and grain refinement. During FSW, the SZ was subjected to extensive plastic deformation, resulting in dynamic recrystallization and the formation of fine equiaxed grains (Figure 5d and Figure 6d). Grain refinement enhanced grain boundary strengthening, but the reduction in deformed structures due to dynamic recrystallization weakens work hardening, resulting in a slightly lower microhardness in the SZ compared to the BM. Additionally, due to the rapid cooling rate during welding, no significant intragranular precipitates were observed, while a small amount of discontinuous T-phase precipitated along grain boundaries (Figure 7d). Compared to the BM, the SZ exhibited a higher quantity and larger size of grain boundary precipitates. These coarse T-phase particles also contributed to the slightly lower microhardness of the SZ.

In the TMAZ, partial deformation occurred during the FSW process, leading to grain bending and dynamic recovery under the thermo-mechanical effect. This resulted in a reduction in deformed structures (Figure 5c and Figure 6c) and weakened work hardening. During welding process, the peak temperature in the TMAZ typically ranges between 280 °C and 320 °C [37]. Under this thermal cycle, T-phase precipitates form at grain boundaries and within grains. In the HAZ, only the thermal cycle of welding is applied [38]. Although the grain morphology remains largely unchanged compared to the BM, this region experienced static recovery. This process reduced the density of deformation structures, thus weakening the work-hardening capacity (Figure 5b and Figure 6b) [39,40,41]. Additionally, coarse T-phase particles precipitate extensively in the HAZ due to the thermal cycle (Figure 7b), further reducing its microhardness. Based on the microstructural characteristics and microhardness tests of the welded joint, the lowest microhardness is observed in the SZ, leading to the tensile samples of the as-welded joint fractured in the SZ.

After aging treatment at 90 °C/24 h + 115 °C/15 h, a high density of nano-scale T-phase precipitate, with sizes ranging from 5 to 10 nm, was formed within the grains in the BM, SZ, and TMAZ of the aged joint. This is identified as the primary reason for the significant increase in microhardness in these regions and the overall strength of the joint after aging treatment. Unlike the other regions, the HAZ in the as-welded state was already populated with coarse T-phase precipitates, whereas only a limited number of nano-scale T-phase precipitates formed during aging. Thus, the aging-induced precipitation strengthening in this region was minimal. Consequently, the lowest microhardness values in the aged joint were observed in the HAZ adjacent to the SZ. Nevertheless, tensile specimens did not fracture in this low hardness zone, indicating a clear deviation from the typical fracture behavior of conventional heat-treatable strengthened alloys.

### 4.2. Analysis of Fracture Behavior of the Aged Joints

**Figure 9:**
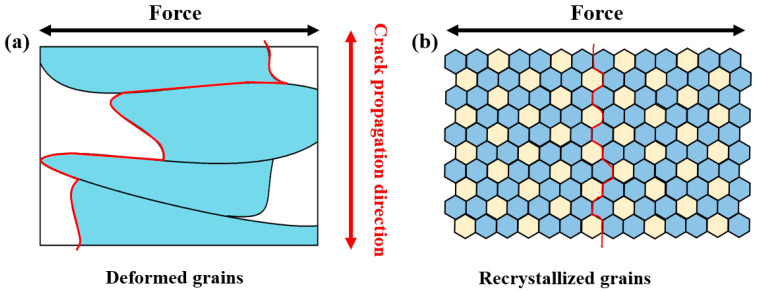
Schematic diagram of fracture mechanisms: (**a**) crack propagation direction in the deformed grains, and (**b**) crack propagation direction in the recrystallized grains.

This section focused on the cause analysis of the abnormal fracture behavior in the aged tensile specimens, aiming to identify the potential microstructural reasons. Fracture analysis of the aged joint (Figure 4) reveals that all tensile specimens failed through intergranular cracking. In the absence of significant welding defects, grain boundary characteristics emerge as the predominant factor governing fracture behavior. During plastic deformation, dislocations accumulate and pile-up at grain boundaries, inducing significant stress concentrations that facilitate crack initiation [42,43]. Precipitate characterization shows continuously distributed T-phase along grain boundaries in all zones of the joint. The width of PFZ follows: HAZ (280 nm) > BM (162 nm) > TMAZ (86 nm) > SZ (81 nm). Wider PFZs facilitate stress concentration and crack initiation during deformation, while continuous T-phase promotes crack propagation. Additionally, grain morphology significantly affects crack propagation [44,45]. Figure 9 compares crack paths in the elongated (Figure 9a) and equiaxed (Figure 9b) grain structures, which shows that the cracks propagate longer distances in elongated grain regions. Although the aged HAZ is the softest region, its elongated grain structure (similar to Figure 9a) resists crack propagation better than the equiaxed grains in the harder SZ (Figure 9b). This explains why tensile specimens consistently fracture in the SZ despite its higher hardness [46]. Optimizing grain boundary precipitates into discontinuous distributions could further improve joint mechanical properties.

## 5. Conclusions

This present work employed a novel high-Mg-containing Al-Mg-Zn-Si alloy for friction stir welding and investigated the microstructure and mechanical properties of the as-welded and aged joints, aiming to investigate the influence of mechanisms of the microstructures in different regions on mechanical properties. The following conclusions were obtained:

1. In the as-welded condition, the microhardness profile of the welded joint exhibited a “W” shape with overall low hardness values and minor differences between zones. After aging treatment, the microhardness increased significantly in the BM, TMAZ, and SZ, whereas the HAZ adjacent to the SZ exhibited only a marginal increase, making it the softest region in the aged joint.
2. The average yield strength, ultimate tensile strength, and elongation of the as-welded joint were 256 MPa, 395 MPa, and 11.20%, respectively. After aging treatment at 90 °C/24 h + 115 °C/15 h, the average yield strength and ultimate tensile strength increased to 327 MPa and 471 MPa, respectively, while the elongation decreased to 5.45%.
3. The enhancement in microhardness and strength after aging is attributed to the precipitation of numerous nano-sized T-phase particles within grains.
4. The aged joint exhibited brittle fracture characteristics due to the presence of continuous grain boundary precipitates, which weakened intergranular cohesion. In contrast to the equiaxed grains in the SZ, the elongated grains in the HAZ more effectively impeded intergranular crack propagation, which explains why the fracture occurred in the high-hardness zone of SZ rather than the low-hardness zone of HAZ.

## Data Availability

The original contributions presented in this study are included in the article. Further inquiries can be directed to the corresponding author.
